# Reevaluating the Role of *Acanthamoeba* Proteases in Tissue Invasion: Observation of Cytopathogenic Mechanisms on MDCK Cell Monolayers and Hamster Corneal Cells

**DOI:** 10.1155/2013/461329

**Published:** 2013-01-01

**Authors:** Maritza Omaña-Molina, Arturo González-Robles, Lizbeth Iliana Salazar-Villatoro, Jacob Lorenzo-Morales, Ana Ruth Cristóbal-Ramos, Verónica Ivonne Hernández-Ramírez, Patricia Talamás-Rohana, Adolfo René Méndez Cruz, Adolfo Martínez-Palomo

**Affiliations:** ^1^UIICSE Faculty of Superior Studies Iztacala, Medicine, UNAM, Los Reyes Iztacala, 54090 Tlalnepantla, MEX, Mexico; ^2^Department of Infectomics and Molecular Pathogenesis, Center for Research and Advanced Studies, 07360 Mexico City, DF, Mexico; ^3^University Institute of Tropical Diseases and Public Health of the Canary Islands, University of la Laguna, Tenerife, Canary Islands, 38200 La Laguna, Spain

## Abstract

The morphological analysis of the cytopathic effect on MDCK cell monolayers and hamster cornea and qualitative and quantitative analyses of conditioned medium and proteases were evaluated and compared between two strains of *Acanthamoeba * genotype T4. Further than highlighting the biological differences found between both strains, the most important observation in this study was the fact that proteases both in total extracts and in conditioned medium are apparently not determinant in tissue destruction. An interestingly finding was that no lysis of corneal tissue was observed as it was previously suggested. These results, together with previous studies, allow us to conclude that the invasion and disruption of corneal tissue is performed by the penetration of the amoebae through cell junctions, either by the action of proteases promoting cellular separation but not by their destruction and/or a mechanical effect exerted by amoebae. Therefore, contact-dependent mechanisms in *Acanthamoeba * pathogenesis are more relevant than it has been previously considered. This is supported because the phagocytosis of recently detached cells as well as those attached to the corneal epithelium leads to the modification of the cellular architecture facilitating the migration and destruction of deeper layers of the corneal epithelium.

## 1. Introduction

Free-living amoebae of the genus *Acanthamoeba* are one of the most common amoebae found in a wide variety of habitats, ranging from tropical zones to arctic regions [1]. These amoebae can be found in dust [2], air, soil [3], fresh water, sea water, tap water [4, 5], bottled mineral water [6], and sewage [7].

These opportunistic pathogens have gained medical importance due to their ability to infect the skin, brain, and eye [8–10]. Various species of *Acanthamoeba* genus can cause granulomatous amoebic encephalitis (GAE), which is usually associated with immunocompromised individuals, and they are also the etiological agent of *Acanthamoeba* keratitis (AK), a painful chronic inflammatory disease of the cornea frequently associated with contact lens wearers [11]. Unlike debilitated patients with GAE or cutaneous acanthamebiasis, individuals with AK are generally immunocompetent. Nevertheless, these individuals do not develop protective immunity, and reinfection can occur. In addition, the infection is highly resistant to many antimicrobial agents mainly due to the existence of a cyst stage in these pathogens [12]. 

At least eight species of *Acanthamoeba* have been implicated in human infections: *A. castellanii *and* A. polyphaga *are the most common isolated species. Molecular classification of* Acanthamoebae* strains has allowed clustering of these pathogens into 17 different genotypes being T4 the most prevalent in environment and clinical cases [13]. 

Parasitic infections may occur in a sequential manner and are initiated by the adherence of the amoebae to the host cells [14, 15]. Amebic adhesion may be mediated by mannose recognition sites localized in the target cells. The recognition of these surface oligosaccharides by *Acanthamoeba* is mediated by a 136 kDa-mannose-binding protein (MBP) on their surface [16]. After recognition and binding, *Acanthamoeba* cytopathogenicity occurs and may result in phagocytosis or induction of host cell necrotic and apoptotic death.

Recently, an *in vitro* animal model of AK has been implemented which allows the evaluation of the early most evident morphological events that take place in the cornea in the target cells. Moreover, it has also been demonstrated that if *Acanthamoeba* trophozoites are cocultured with isolated hamster and human corneas, the amoebae are able to invade and cause damage to the intact corneal epithelium, without the requirement of a previous corneal abrasion [17, 18].

The role of proteases in these processes has been previously discussed [13]; however, it has been evaluated in cellular monolayers and not directly in the target tissue. For that reason it is important to determine the role of these proteases, the phagocytosis phenomena and the mechanical action that these amoebae exert on the target tissue during the invasion process.

## 2. Material and Methods

### 2.1. Amoebae

This study was carried out with two *Acanthamoeba* strains isolated in the association to prevent blindness in Mexico, (Luis Sánchez Bulnes Hospital, Mexico City); *Acanthamoeba polyphaga *was obtained from the contact lens of a patient with AK, and *Acanthamoeba castellanii* was also isolated from the contact lens of a patient that was suffering intense ocular pain. No amoebae were isolated from the corneal scrapes of these patients. Amoebae were preliminarily identified to the species level using the morphological criteria of [20]. Molecular identification of the amoebic strains at the genotype level was carried out as previously described by sequencing the diagnostic fragment 3 (DF3) of the 18S rDNA gene of *Acanthamoeba* [20, 21].

### 2.2. Isolation and Maintenance of *Acanthamoeba* Strains in Monoxenic Cultures

The techniques used for recovery and maintenance of *Acanthamoebae *from clinical and environmental sources are described elsewhere [22, 23]. Briefly, primary isolation was performed from infected human corneal tissues and contact lenses using 1.5% nonnutrient agar plates seeded with heat-killed *Enterobacter aerogenes*. Subsequent incubation was performed at ambient temperature (22 to 24°C) for up to 10 days. Upon evidence of amoebic growth, clonal cultures were established by transferring a single double-walled cyst to fresh agar medium containing antibiotics (penicillin, 100 mg/mL; streptomycin, 10 mg/mL). 

### 2.3. Axenic Cultures

Monoxenic cultures were selected from areas of profuse amoebic growth. Selected pieces of agar were transferred to axenic culture mediums such as phosphate-biotriptase-serum glucose medium (PBSGM) [24] and 2% Bacto Casitone medium (DIFCO), which are culture media widely used for growth and amoebic development. Both mediums were supplemented with 10% fetal bovine serum (Equitech-bio, Kerville, TX, USA) and 1% of an antibiotics mix (Penicillin-Streptomycin). Trophozoites were incubated in both mediums at 30°C in borosilicate tubes (Pyrex, Mexico). The medium was changed twice daily for 2 days and afterwards once daily for 3 more days. The cultures were considered axenic if no bacterial growth was observed. It is important to emphasize that all trophozoites interactions assays were performed with Bacto Casitone medium free of fetal bovine serum.

### 2.4. Temperature Tolerance Test

To determine the optimal culture medium and temperature for growth, amoebae were incubated at 30°C, 35°C, and 37°C in borosilicate tubes (Pyrex, Mexico). Assays were carried out simultaneously by placing axenic trophozoites in the culture media mentioned above. Optimal growth was determined by plotting logarithmic growth phase curves (triplicate assays were performed). The viability of the trophozoites was determined using the trypan blue (0.4%) exclusion test. 

### 2.5. Pathogenicity Test

Trophozoites from axenic cultures in the exponential phase of growth (2.5 × 10^5^ parasites/20 *μ*L) were inoculated into the nostrils of 15 male Balb/C mice (3 weeks old), according to Culbertson et al. [19]. A group of five mice was inoculated with culture medium without amoebae used as control. After 21 days, the surviving mice were sacrificed. The brain, liver, lungs, and kidneys were cultured in agar plates with nonnutritive enriched medium (NNE) to recover the amoebae [24, 25].

### 2.6. Quantitative Analysis of Trophozoite Adherence to MDCK Cells

Monolayers of epithelial cells of the established MDCK line of canine kidney origin (Madin Darby Canine Kidney) were grown on 25 cm^2^ cell culture flasks (Corning Incorporated, NY) in Dulbecco's modified Eagle's medium (Microlab, Mexico). They were supplemented with 10% fetal bovine serum (Gibco, Grand Islands, NY) and antibiotics in a 5% CO_2_ atmosphere at 37°C.

Cells were trypsinized and transferred to 96 well plates. Confluent cell monolayers were fixed with 2.5% glutaraldehyde in 0.1 M cacodylate buffer for 1 h and washed twice with Dulbecco's Phosphate Buffered Saline (DPBS). Trophozoites from both *Acanthamoeba* species (7.5 × 10^4^) were added to the monolayers and incubated at different times (5, 10, 15, 30, 45, 60, 120, and 180 min) at 30°C, and fixed as mentioned above. Then samples were washed twice with DPBS-Tween 0.05%. Adhesion was evaluated by an ELISA-based assay; briefly the plates were blocked overnight with 200 *μ*L of 1% casein at 4°C, and plates were washed 5 times with PBS-Tween 0.05% and 100 *μ*L of polyclonal IgG anti-*Acanthamoeba*: *A. castellanii* (1 : 8000) and *A. polyphaga* (1 : 4000) were added and incubated for 2 h at 37°C. Subsequently, plates were washed 5 times with PBS-Tween then 100 *μ*L of a dilution of 1 : 50000 conjugate anti-rabbit IgG was added and incubated for 1 h at 37°C. After that 100 *μ*L of developing solution was added for 10 min; at that moment a mixture of citric acid and 0.1 M dibasic sodium phosphate 0.2 M pH 5, finally 40 mg of orthophenylenediamine and 40 *μ*L hydrogen peroxide 30% were added. The reaction was blocked with 50 *μ*L of H_2_SO_4_ to 2.5 M. Absorbance was read at 490 nm (Bio-Rad 550) [26].

### 2.7. Cytopathic Effect on MDCK Cells

MDCK cell monolayers were trypsinized and transferred to round plastic cover slips placed in 24 well styrene plates and kept at 37°C in 5% CO_2_; after 24 h cells formed confluent monolayers. Afterwards, cell cultures were incubated at different times (1, 2, and 3 h) in the same conditions in a mixture of amoebae medium and Dulbecco's modified Eagle's medium serum free (Gibco BRL) in equal proportions. *A. castellanii *and* A. polyphaga *trophozoites were added in a 1 : 1 (target cell: amoebae) ratio.

### 2.8. Scanning Electron Microscopy

Samples were fixed with 2.5% glutaraldehyde in 0.1 M cacodylate buffer, and dehydrated with increasing concentrations of ethanol. Samples were then critical point-dried with liquid CO_2_ using a Samdri 780 apparatus (Tousimis Research Corp.) and coated with a thin layer (30 nm) of gold in an ion-sputtering device (JEOL, JFC-1100). Specimens were examined with a Philips XL30 ESEM scanning electron microscope. 

### 2.9. Interactions with Hamster Cornea

Adult male golden hamsters (*Mesocricetus auratus*) weighing 120 to 130 g were used. Experiments were based on protocol 002/02, approved by the Institutional Animal Care and Use Committee, in accordance with norm-062-Zoo-1999, based on the *Guide for the Care and Use of Laboratory Animals*, published in the Official Journal of the Federation (Mexico) 2001. After anesthesia with sodium pentobarbital (Sedatphorte) at 4.72 mg/100 g of body weight, both corneas were removed leaving a peripheral rim of scleral tissue, as previously described [12]. Corneas were placed in 96 well plates and interacted with 2.5 × 10^5^ trophozoites for different periods of time (30 min, 1, 2, 4, 8, 16, and 24 h). Control corneas were treated in a similar way, but only culture medium was added.

### 2.10. Interactions of Conditioned Medium with Hamster Cornea

Assays were carried out as described above by using conditioned medium obtained as follows: 2 × 10^5^ trophozoites from a culture in exponential phase of growth were placed in culture flasks containing 5 mL of fresh Bacto Casitone or PBSGM mediums free of fetal bovine serum and incubated at 30°C for 24 h. Trophozoites were then chilled on ice for 10 min and centrifuged. The supernatant was removed, centrifuged at 1500 g, and filtered through 0.22 *μ*m filters (Millex GV Durapore PVDF). Viability of trophozoites was determined using the trypan blue exclusion technique before collecting conditioned medium [27].

### 2.11. Light and Transmission Electron Microscopy

 After coincubation, samples were fixed with 2.5% glutaraldehyde in 0.1 M cacodylate buffer, pH 7.2 at room temperature, postfixed with 1% osmium tetroxide, and dehydrated with increasing concentrations of ethanol. Samples were transferred to propylene oxide, later on to a mixture of propylene oxide/epoxy resin (1/1) and finally embedded in epoxy resins. Semithin sections (0.5 *μ*m), stained with toluidine blue were examined with an Axiophot photomicroscope (Carl Zeiss, Germany). Thin sections previously stained with uranyl acetate and lead citrate were observed in a Morgagni 268 D transmission electron microscope (FEI Company, Eindhoven, The Netherlands). 

### 2.12. Whole-Cell Extracts and Conditioned Medium of *A. castellanii* and *A. polyphaga*

*Acanthamoeba* spp. trophozoites in logarithmic phase of growth were chilled at 4°C, centrifuged and washed twice with DPBS. The cells were disrupted by 10 vortex-ice cycles in DPBS. Protein concentration (1 × 10^5^ trophozoites) was quantified by Bio-Rad RC-DC method. 

The conditioned media from both species were obtained as follows: 6 × 10^6^ trophozoites from a culture in exponential phase of growth were placed in culture flasks containing 5 mL of fresh Bacto Casitone free of fetal bovine serum and incubated at 30°C for 24 h. The viability of the amoebae was determined using trypan blue exclusion test. Trophozoites were then chilled on ice for 10 min, and centrifuged. The supernatant was removed, centrifuged, and filtered through 0.22 *μ*m filters (Millex GV Durapore PVDF). Total crude extracts and conditioned media were stored at −70°C until used.

### 2.13. Protease Inhibitors

The inhibitors concentrations used were as follows: N-ethylmaleimide (NEM, cysteine proteases inhibitor) 20 and 50 mM; ethylenediaminetetraacetic acid (EDTA, metalloproteases inhibitor) 100 and 200 mM; phenylmethylsulfony fluoride (PMSF, serine proteases inhibitor) 1 and 5 mM.

### 2.14. Substrate Gel Electrophoresis

To detect proteolytic activity in crude extracts and conditioned medium, SDS-PAGE gels were copolymerized with 0.1% (w/v) gelatin as protease substrate. 

The electrophoresis was performed at 4°C for 1 h; gels were washed twice with 2.5% triton X-100 solution and incubated at 37°C overnight with 100 mM Tris-OH buffer (pH 7.0) supplemented with 2 mM CaCl_2_. Gels were stained with 0.5% Coomassie blue R-250 [28].

## 3. Results

### 3.1. Axenic Cultures and Optimal Temperature of Growth

**Figure 1:**
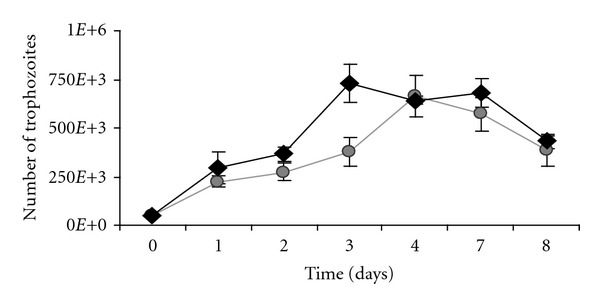
Representative growth curves of the optimal temperature (30°C) for *A. castellanii *(grey circle) in bacto casitone medium and for *A. polyphaga *(black diamond) in PBSG medium. Assays were carried out in triplicate with 98% parasite viability.

*Acanthamoeba castellanii* grew better in Bacto Casitone medium while the best growth rate for *Acanthamoeba polyphaga* was observed in PBSG medium. The optimal growth temperature for both amoebae was 30°C. *A. polyphaga* reached the exponential phase of growth in 48–72 h, with a homogeneous and abundant number of trophozoites that were able to form mature cysts after several weeks. In contrast, *A. castellanii* reached the exponential phase of growth after 72–96 h of culture. The cultures had a low number of trophozoites and form mature cysts in a few days (Figure 1). After sequencing of the DF3 region of both strains, it was concluded that both amoebae belonged to genotype T4.

### 3.2. *A. castellanii* Is Virulent in Animal Model of EAG in Contrast with *A. polyphaga*

It is only invasive. Mice infected with *A. castellanii* became ill very quickly, as it was manifested by ruffled fur and aimless wandering of the animals, followed by coma and death 5 to 9 days after inoculation. Amoebae were isolated from the brain, liver, lung, and kidney. In contrast, mice infected with *A. polyphaga *showed little evidence of illness. Only 3 mice died between 7 to 20 days after inoculation, and amoebae were recovered only from the brain and lung (Table 1).

### 3.3. Trophozoites of *A. castellanii* and *A. polyphaga* Showed Significant Differences in Their Adhesion Rate to MDCK Cells

**Figure 2:**
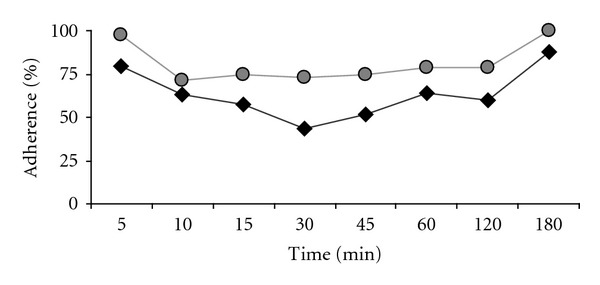
Adherence of *Acanthamoeba* spp. to MDCK cells. Trophozoites (7.5 × 10^4^) of *A. castellanii *(grey circle) and *A. polyphaga *(black diamond) were incubated for different times with confluent MDCK epithelial cell monolayers. Adhesion was evaluated by an ELISA-based assay (absorbance of 7.5 × 10^4^ trophozoites corresponded to 100%). Assays were performed in triplicate.

Significant differences in the adhesion rate to MDCK cells were observed.  Figure 2 shows the percentage of adherence of amoebae to monolayers of MDCK cells at different times of interaction. Adherence of *A. castellanii* was higher at all time when compared to the *A. polyphaga* strain. Roughly 90% of *A. castellanii* trophozoites adhered to the monolayer after 5 min of incubation, while less than 80% of *A. polyphaga* trophozoites were able to adhere to the cells. Nevertheless, adherence in both strains was time dependent, reaching 100% adherence after 180 min.

The obtained data were transformed and analyzed as their natural logarithm as they were not normally distributed (Kolmogorov-Smirnov test). A two-factor analysis of variance (ANOVA) showed significant differences in the adhesion of the two species under study, as well as differences along the interaction time (*P* < 0.001).

### 3.4. Trophozoites of *A. castellanii* Individually or in Groups Were Observed Penetrating the MDCK Monolayer Forming Protuberances on It

**Figure 3:**
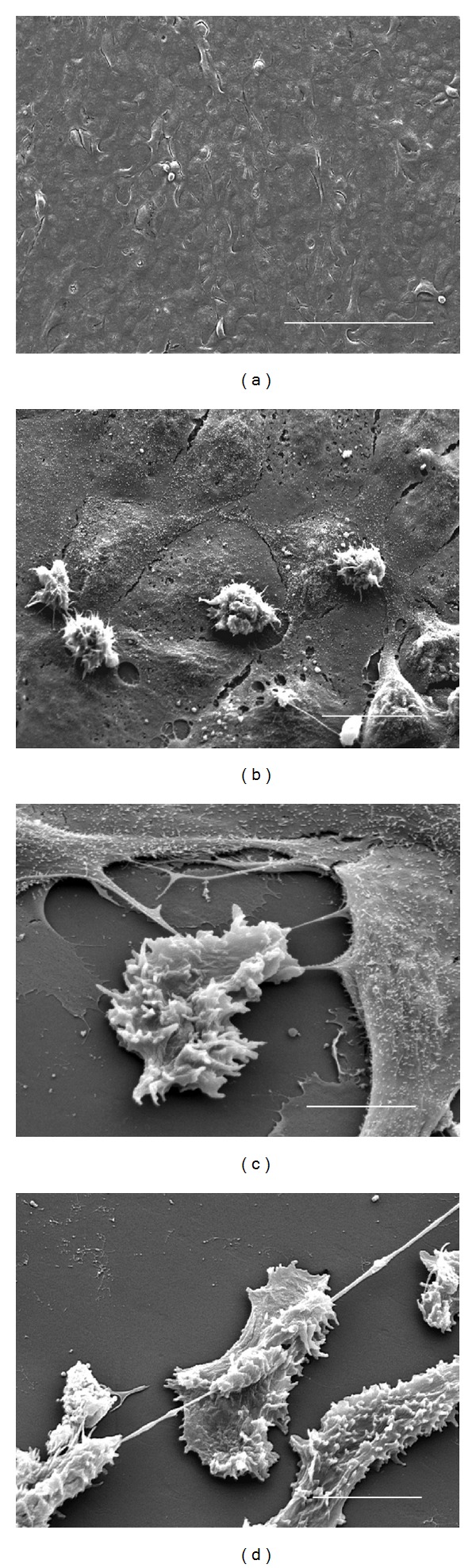
Scanning electron microscopy of the interaction of *A. polyphaga* with MDCK cells. (a) No morphological evidence of damage was observed on control MDCK monolayers incubated for 3 h with 1 : 1 PBSGM : DMEM medium. Bar = 200 *μ*m. (b) After 1 h of coincubation, small number of trophozoites were observed adhered to MDCK cells surface Bar = 20 *μ*m. (c) By 2 h only few areas of monolayer discontinuity were observed, and a small number of trophozoites were located under and over the surface of MDCK cells. Bar = 10 *μ*m. (d) At 3 h of coincubation, scarce areas devoid of cells were observed, in which a trophozoite was seen phagocyting fragments of MDCK cells. Bar = 10 *μ*m.

**Figure 4:**
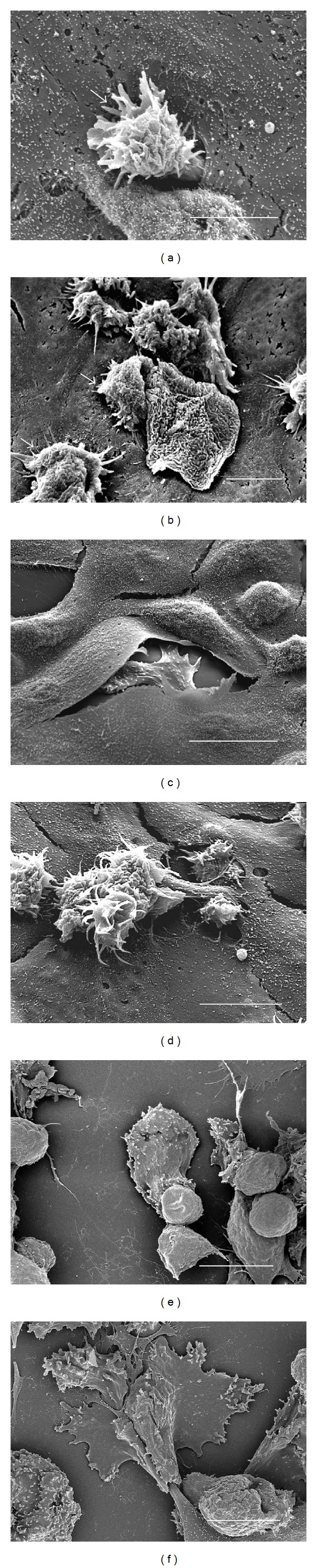
Scanning electron microscopy of the interaction of *A. castellanii* with MDCK epithelial cell monolayer. After 1 h of interaction, alone (a) or in group (b) trophozoites were observed penetrating the MDCK monolayer. Typical acanthopodia were clearly seen (arrows). Bar = 10 *μ*m. (c) In another area one trophozoite penetrated the monolayer and formed a protuberance on the surface. (d) By the second hour, numerous trophozoites were seen adhered to the monolayer apparently phagocyting a MDCK cell. Bar = 10 *μ*m. (e) Clearly damaged areas of the cell monolayer were observed 3 h after interaction. Several regions of the substrate became apparent as a consequence of the detachment and/or ingestion of the MDCK cells by the amoebae. Bar = 20 *μ*m. (f) Trophozoite apparently phagocyting a portion of an MDCK cell. Bar = 20 *μ*m.

No morphological evidence of damage was observed in the MDCK control monolayer after 3 h of interaction (Figure 3(a)). At 1 h of interaction only a small number of *A. polyphaga* trophozoites penetrated the monolayer (Figure 3(b)), scarce areas devoid of cells (Figure 3(c)) and trophozoites phagocyting minor fragments of MDCK cells were observed at 3 h (Figure 3(d)). In contrast, from the first hour of interaction, trophozoites of *A. castellanii* individually or in groups were observed penetrating the MDCK monolayer (Figures 4(a) and 4(b)), forming protuberances on it (Figure 4(c)). By the second hour, trophozoites were seen adhered and beneath to the monolayer apparently phagocyting MDCK cells (Figure 4(d)). Several regions of the substrate became apparent as a consequence of the detachment and/or ingestion of the MDCK cells by the amoebae and during all assays parasites apparently ingested whole cells or fragments of detached cells (Figures 4(e) and 4(f)). Intact MDCK cells were observed in close proximity to the damaged areas, suggesting a focal damage of the cell monolayer related to a contact-dependent trophozoite-target cell interaction.

### 3.5. Structural Analysis of the Interaction Revealed That Both Amoebae Produced Different Degrees of Damage to the Corneal Epithelium

**Figure 5:**
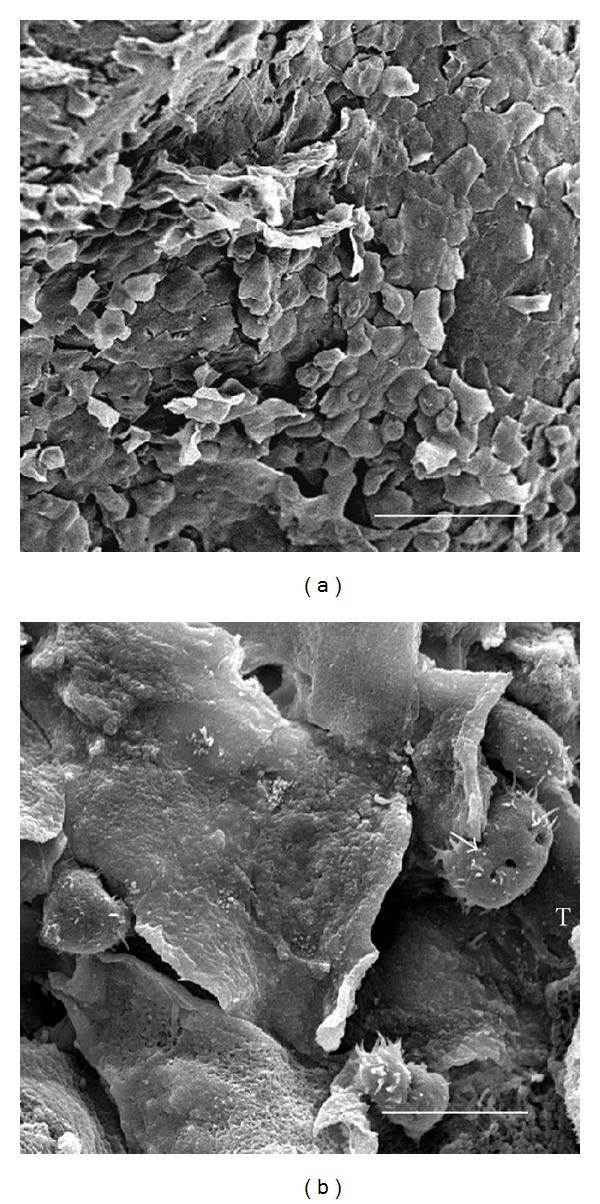
Scanning electron microscopy of the interaction of *A. polyphaga* trophozoites with hamster cornea. In the first hours of coincubation areas of corneal injury were not observed. (a) After 16 h, only few regions of cellular disorganization and loss of the most superficial layers of the cornea were detected. Bar = 100 *μ*m. (b) Trophozoite (T) was found in close relation with epithelial cells (arrow). Bar = 10 *μ*m.

**Figure 6:**
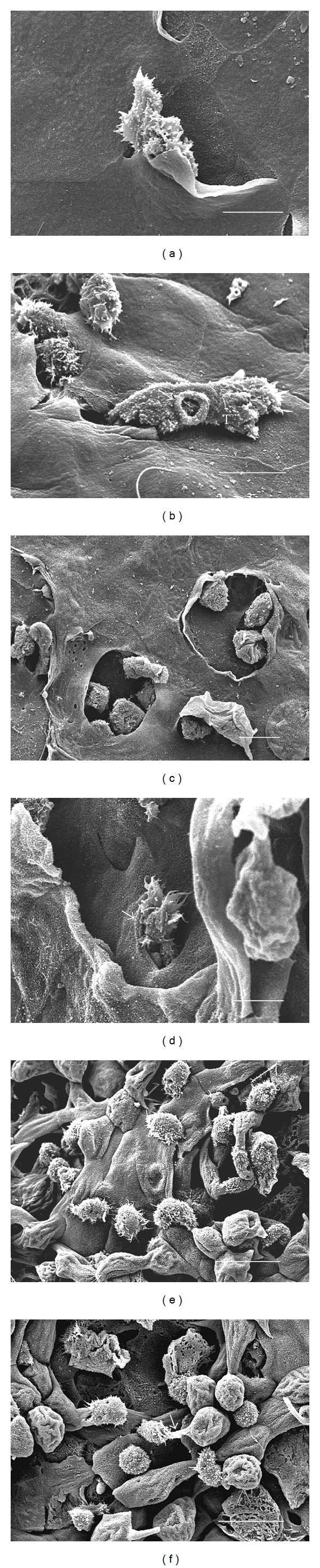
Scanning electron microscopy of the interaction of *A. castellanii* trophozoites with hamster cornea. (a) At 1 h, one trophozoite was observed penetrating the most superficial epithelial cells. Bar = 10 *μ*m. (b) Trophozoite (T) emitting a phagocytic structure (arrow) was a frequent event. Bar = 10 *μ*m. (c) After 2 h of interaction numerous trophozoites were located under the first layer of the corneal epithelium. Bar = 20 *μ*m. (d) By 4 h *A. castellanii* continued migrating towards the deepest layers of the corneal epithelium (arrow). Bar = 10 *μ*m. (e) At 16 h, numerous trophozoites remained adhered to the surface of the epithelial cells that had not been detached. Bar = 20 *μ*m. (f) After 24 h damage to the structure of the cornea is evident. A trophozoite was observed apparently phagocyting an epithelial cell (arrow). Bar = 20 *μ*m.

After 16 to 24 h of interaction *A. polyphaga* reached only the most superficial corneal cells (Figure 5(a)). Few trophozoites migrated to wing cells layer phagocytizing detached cells (Figure 5(b)). In contrast,* A. castellanii* invaded and disorganized corneal epithelium by penetrating through the cell junctions into the inner epithelium layers (Figure 6(a)), emitting phagocytic structures (Figure 6(b)). At 2 h, trophozoites were seen beneath the superficial cell layers detaching epithelial cells (Figure 6(c)). From 4 h to 16 h, trophozoites continued migrating towards the deepest layers of corneal tissue (Figure 6(d)), which had lost its original morphology at this stage. Moreover it was not possible to recognize in which region of the epithelium the trophozoites were located due to the damage caused at this stage (Figure 6(e)). After 24 h of interaction, the migration of the trophozoites continued and phagocytosis was constantly observed (Figure 6(f)).

### 3.6. Semithin Sections of the Squamous Epithelium Show Focal Damage Related to Contact-Dependent *A. castellanii* Trophozoite-Target Cell Interaction

**Figure 7:**
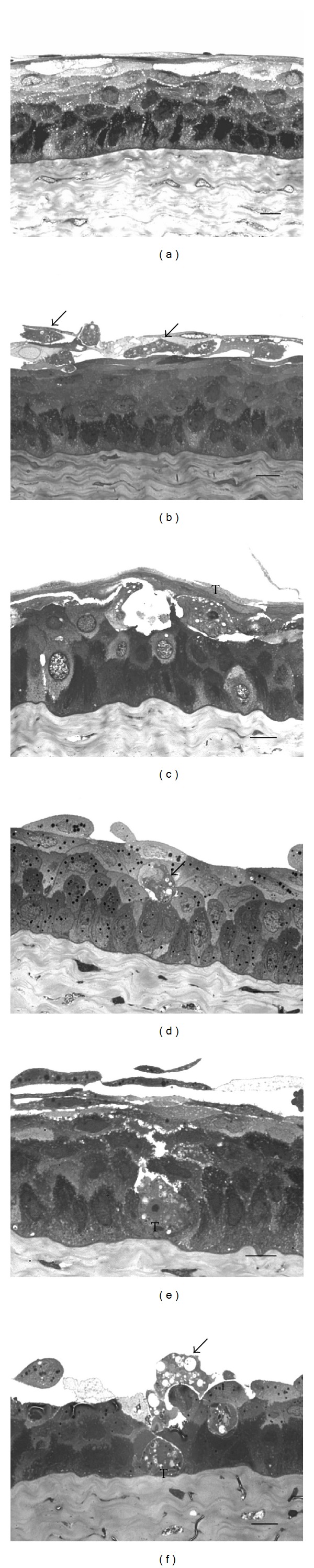
Light microscopy of semithin sections of the interaction of *A. castellanii* trophozoites with hamster cornea. The most representative time points are shown. (a) Control hamster cornea incubated with Bacto Casitone medium for 24 h. No evidence of damage was observed. (b) After 1 h of interaction numerous trophozoites (arrows) were observed penetrating the most superficial layers of hamster's cornea. (c) After 8 h a trophozoite (T) migrated toward wings epithelial cells; part of the corneal epithelium remained, but the normal tissue structure had been lost. (d) At 16 h superficial and some middle (wings) cells of the corneal epithelium had been separated from the rest of the tissue. A trophozoite phagocyting an epithelial cell is shown (arrow). (e) After 24 h of interaction *A. castellanii* trophozoite had migrated toward the basal cells. (f) In other areas of interaction most of the epithelium had been lost. A trophozoite (T) reached the corneal stroma layer while another was seen phagocyting a detached cell (arrow). Bar = 10 *μ*m.

Semithin sections of the squamous epithelium of control corneas incubated in Bacto Casitone culture medium showed a typical morphology as well epithelium incubated with PBSG medium (Figure 7(a)).

During early interaction times, numerous trophozoites were observed penetrating the most superficial corneal epithelial layers (Figure 7(b)). By 8 h trophozoites had migrated toward wings epithelial cells (Figure 7(c)). At 16 and 24 h of interaction *A. castellanii* migrated and injured basal epithelial corneal cells (Figures 7(d) and 7(e)) and stroma layer (Figure 7(f)). Areas in which the amoebae attached and penetrated were morphologically intact suggesting a focal damage related to a contact-dependent trophozoite target cell interaction. Phagocytosis was a recurrent process (Figures 7(d) and 7(f)). No evidence of harm produced by *A. polyphaga* trophozoites was seen at the same time of incubation. 

### 3.7. *A. castellanii* Invade Tissue with Acanthopods and Phagocyte Epithelial Corneal Cells with Amoebostomes

**Figure 8:**
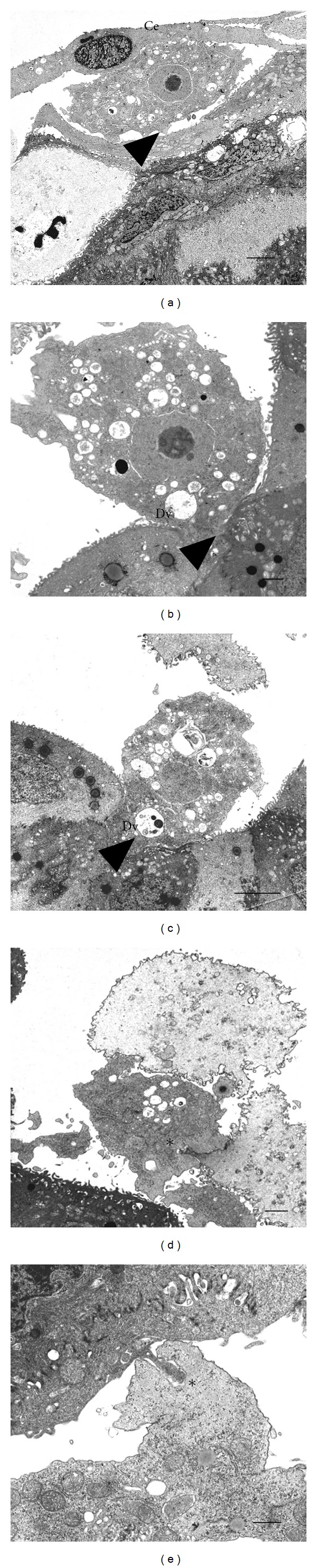
Transmission electron microscopy of the interaction of *A. castellanii* with hamster cornea. (a) After 16 h of interaction a trophozoite migrated and penetrated into middle layer of corneal epithelium (Ce), emitting acanthopodia of different sizes (arrow head). Bar = 3 *μ*m. (b) and (c) By 24 h trophozoites migrated to deeper layers presumably by the emission of cytoplasmic projections (arrow head), which were frequently observed being introduced between the epithelial cells junctions. The amoebae preserved their characteristic morphological features. Some trophozoites shown in the figure contain digestive vacuoles (Dv) of different sizes. (b). Bar = 1 *μ*m; (c). Bar = 3 *μ*m. (d) and (e) Frequently, amoebae emitted amoebostomes (∗) of different sizes with their characteristic fibrogranular cytoplasm, which allowed them to ingest portions of cells. (d). Bar = 1 *μ*m; (e). Bar = 1 *μ*m.

Invasion by amoebae takes place with cell projections (acanthopodia) which were frequently observed (Figure 8(a)), allowing the passage of amoebae between corneal epithelial cells junctions (Figures 8(b) and 8(c)). Amoebae regularly emitted amoebostomes of different sizes with their characteristic fibrogranular cytoplasm ingesting whole cell or portion of them (Figures 8(d) and 8(e)). Digestive vacuoles were a frequent finding. 

### 3.8. Conditioned Medium Does Not Lyse Hamster Cornea Tissue

**Figure 9:**
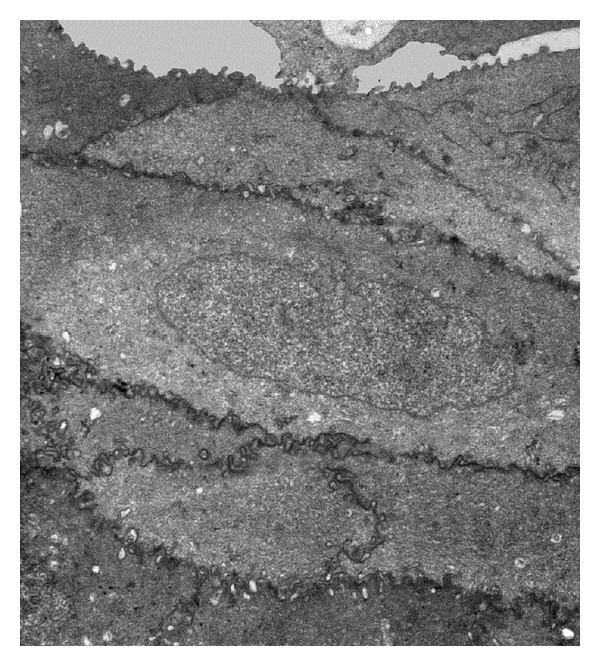
Interaction of conditioned culture medium with hamster cornea. At 6 h after interaction with Bacto Casitone medium, no evidence of damage or cell disorganization was observed; only normal desquamation was detected in scarce zones of corneal epithelial surface.

Analysis of the conditioned culture medium interaction with hamster cornea at different point times showed that neither Bacto Casitone nor PBSGM conditioned mediums were able of lysing cells of corneal epithelium by itself, since no evidence of damage or cell disorganization was observed (Figure 9).

### 3.9. Proteolytic Activity of Whole-Cell Extracts of *A. castellanii* and *A. polyphaga* with Conditioned Medium

**Figure 10:**
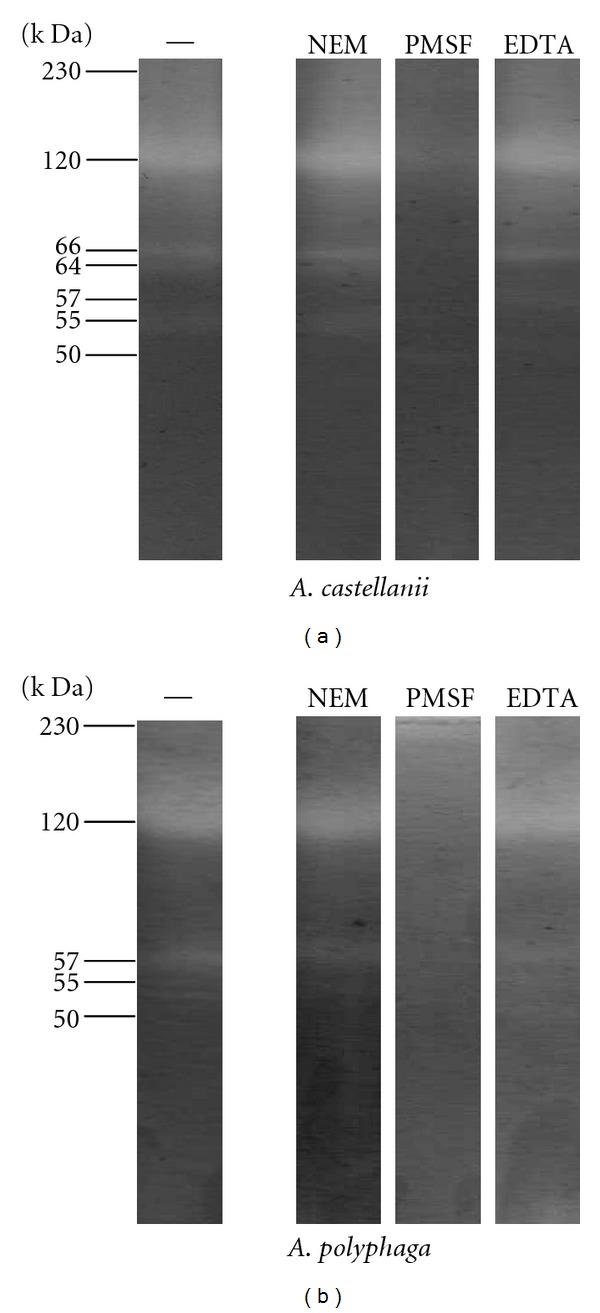
Proteolytic activity profiles from *A. castellanii* and *A. polyphaga*. 1 *μ*g of protein was analyzed from total extracts, which is equivalent to 3.6 × 10^3^ trophozoites. The extracts were obtained from axenic cultures of *A. castellanii* and *A. polyphaga*; these were analyzed on SDS PAGE gels copolymerized with gelatin and incubated in the presence of protease inhibitors with the following concentrations: 20 mM NEM, 100 mM EDTA, and 1 mM PMSF. Protease analysis represents the mean of 5 separate experiments.

The profiles of proteolytic activity of extracts from *A. castellanii* and *A. polyphaga *showed the presence of constitutive proteases in both strains (Figure 10). In the case of *A. castellanii* proteases were 64, 66, and 120 kDa molecular weight. In comparison, *A. polyphaga* presents proteolytic activities ranging between 55, 57, and 120 kDa. 

Proteolytic activity inhibition tests strongly suggest that both amoebae produce serine proteases. 

**Figure 11:**
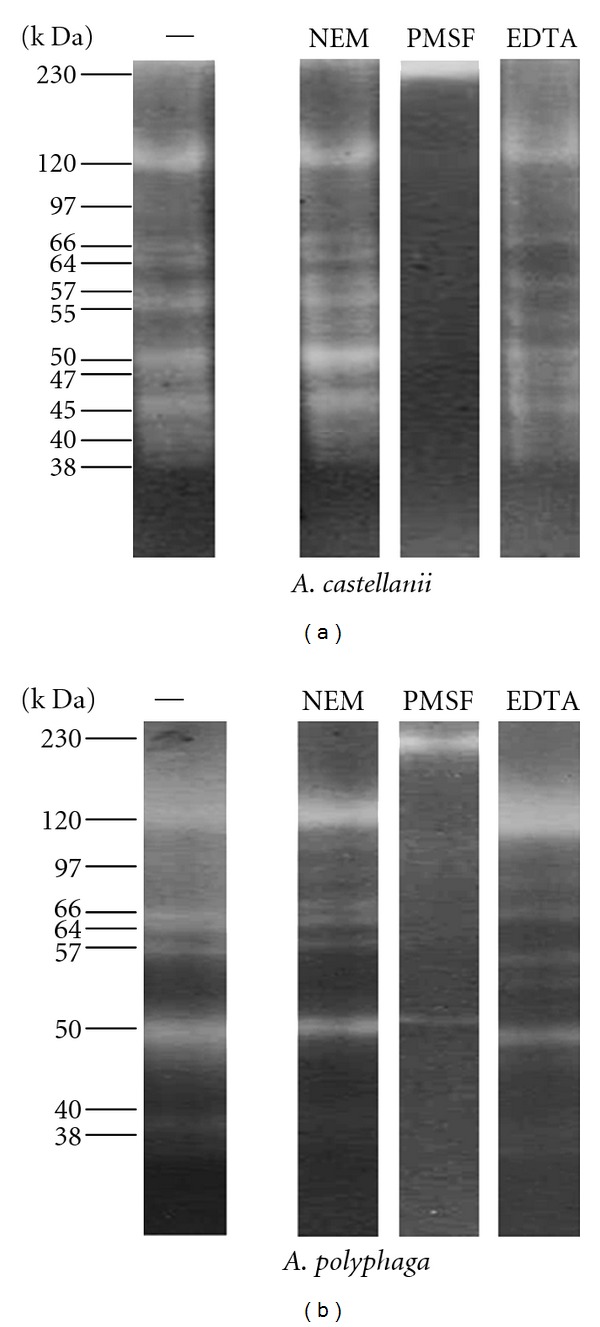
Proteolytic activity of conditioned medium of *A. castellanii* and *A. polyphaga*. SDS-PAGE gels copolymerized with gelatin were incubated independently with the following inhibitors: 20 mM NEM, 100 mM EDTA, and 1 mM PMSF. Protease analysis represents the mean of 5 separate experiments.

Analysis of proteolytic activity from the conditioned medium (Figure 11) evidenced the presence of proteases with molecular weight similar to those detected in extracts from trophozoites of both strains. The tests performed with the proteases inhibitors showed again that the main proteases profile belongs to the family of serine proteases, because the PMSF showed a better inhibitory effect.  Table 2 summarizes the molecular weights detected in both conditions. 

## 4. Discussion

*Acanthamoeba* keratitis has been recognized as a significant ocular amoebic infection where use of contact lenses plays an important risk factor [29], as they cause epithelial changes which may reduce the corneal epithelial resistance to microbial invasion [13]. At present, there is little information about differences in biological and cytopathogenic mechanisms between *Acanthamoeba* species, and whether they are related or not to the virulence of each strain. In the course of the analysis of the results, we determined that even though both species in this study were evaluated under the same laboratory conditions and belonged to the same potentially pathogenic genotype (T4); significant differences were found between them. It was observed that *A. castellanii* strain grew slower than *A. polyphaga*. We expected that the fast-growing *A. polyphaga *could facilitate *in vitro* damage in target cells and therefore establish an infection more easily than slow-growing *A. castellanii*, creating enough amoebae to start an infection and therefore growing more rapidly in the host; however, the results showed otherwise. Moreover, significant differences were observed during the tissue invasion of amoebae to mice organs incubated *postmortem*; *A. castellanii* invaded all the evaluated organs (brain, lungs, liver, and kidney) being more virulent and invasive than *A. polyphaga* which was able to invade only brain and lung. These results are in agreement with Levandowsky et al. [30], who reported that pathogenic free-living amoebae migrate more rapidly than nonpathogenic species.

*A. castellanii* shown to be more efficient during all the evaluated processes since the quantitative and qualitative determination of the adherence rate was constant and higher, correlating with migration and penetration to the MDCK monolayer epithelial cell, mice pathogenicity test, and hamster cornea damage.

Phagocytosis plays an important role in the pathogenic mechanisms of *Acanthamoeba* species, since it is the mechanism of food acquisition during this parasitic phase. The loss of stromal keratocytes due to phagocytosis and apoptosis by *Acanthamoeba* has been previously documented through *in vitro* studies [8, 31]. 

As observed in this work, *Acanthamoeba* is able to damage intact corneal epithelium phagocytizing not only the keratocytes that are located in deeper layers of corneal tissue, but also the most superficial cells.

The most important issue in this study is that in addition to showing in detail the process of phagocytosis and migration of amoebae within the cell junctions, it was also possible to evaluate the proteases and their participation in the target tissue damage in a similar way as it takes place *in vivo*.

Zymography assays performed in this work confirmed that almost all proteases in trophozoites of *A. polyphaga* and *A. castellanii *were mainly serine proteases. *A. castellanii* produced eleven extracellular proteases and *A. polyphaga* seven with similar molecular weight, which is in accordance with previous studies with T4 strains. These previous studies have evaluated T4 isolates associated with GAE and AK allowing the identification of extracellular serine proteases of 36, 49, and 66 kDa [28], all of them with similar molecular weight to the ones observed for the strains included in this study. Cao et al. [11] reported 55, 97, and 230 kDa serine proteases, which correlates to the 55 y 97 kDa proteases found in this work. Alfieri et al. [32] reported 27, 47, 60, 75, 100, and lower than 110 kDa serine proteases; showing a coincidence with the 47 y 60 kDa proteases found in our strains. It is also important to mention that a 40 kDa serine protease related to genotype T4 [33] was also observed in our strains corroborating their genotypical classification. Even though the presence of proteases was confirmed in both species, *A. castellanii* secrete most of them. 

Clarke and Niederkorn [34] suggested that pathophysiology of this infection includes the production of several pathogenic proteases that degrade basement membranes and induce cytolysis of the cellular elements of the cornea, conversely, we consider that the process of cornea invasion differs from the previously proposed, since the observed damage on the target tissue was strongly associated with amoeba contact-dependent mechanism more than damage due to independent mechanisms during amoebae corneal epithelium invasion. As mentioned, amoebae individually or grouped penetrate through the cell junctions and the neighborhood does not reveal any damage. Sometimes even when amoebae penetrate deeper in the corneal epithelium, no evidence of complete destruction of corneal tissue was observed. Besides it was not possible to see how trophozoites got to that site, Garner [8] reported similar images in histological sections of patients. We observed comparable results when evaluating early events of the invasion of *Acanthamoeba* trophozoites when introduced intranasally in a mice model for granulomatous amoebic encephalitis (data not shown). Our results are in agreement with Takaoka-Sugihara et al. [31], who suggested that direct contact with trophozoites, but not with soluble factors, was essential to induce the cytopathic effect on human corneal cells.

Proteolytic activity has been considered as a main source of damage, leaving phagocytosis in a second term as well as the mechanical forces produced by the amoebae during migration through the cells.

The results of this study agree that enzymatic contribution is relevant but it is imperative to emphasize that no evidence of whole cell lysis was found during the interaction assays with the MDCK cells and hamster cornea until the phagocytosis process was detected. The results observed in this study show that conditioned medium may promote the separation between the cells, leaving small spaces between them, which may facilitate the passage of amoebae between cell junctions. Besides we have proved that conditioned medium alone is able to damage only the most superficial human corneal tissue but amoebae invade and phagocyte epithelial cells reaching the Bowman layer of human cornea [17]. Ruqaiyyah and Khan [35], with reference to contact-independent factors of *Acanthamoeba,* have mentioned that these amoebae possess hydrolytic enzymes including elastases, phospholipases, glycosidases and a variety of serine, cysteine and metalloproteases, and ensure that their precise mechanisms of action at the molecular level are only beginning to emerge. We propose that the participation of proteases in this process facilitates passage of amoebas through corneal cells either by proteases facilitating cell separation but not their destruction. The findings suggest that intimate contact with the target tissue is important since trophozoites are able to form cytoplasmic projections between one cell to another in order to migrate and invade deeper layers of the target tissue. It could be possible that the movement of amoebae between the cells exerts a mechanical force that promotes separation and allows migration into the deeper layers of corneal tissue and MDCK cells, which may also explain the loss of the cellular structure (without the loss of integrity or damage to corneal cells). We emphasized that enzymatic activity may play a role in the citolytic mechanisms facilitating the separation of cells and probably not by destroying the tissue as it has been previously suggested. 

It has been demonstrated that two species of *Acanthamoeba *genus even belonging to the same genotype showed important differences in their biological and cytopathogenic mechanisms. Takaoka-Sugihara et al. [31] suggested pathophysiological diversity of *Acanthamoeba* within the T4 genotype based in varied cytopathic effect provoked by these amoebae. 

Amoebic pathogenicity may be an intrinsic characteristic and *Acanthamoeba *infection could be a correlation between amoebic features such as growth temperature, adhesion, phagocytosis, proteolytic activity, and mechanical effect during the invasion process as well as the host conditions. 

## 5. Conclusion

Further than showing differences found between amoebae, the most important remark in this study is that the presence of proteases in both, total extracts and in conditioned medium are not determinative in tissue destruction. In view of our results, together with previous studies, we suggest that the invasion and disruption of corneal tissue is performed by the penetration of the amoebae through cell junctions either by the action of proteases only promoting cellular separation but not their destruction and/or a mechanical effect exerted by amoebae. Phagocytosis of recently detached cells as those attached to the corneal epithelium, leads to the modification of its architecture, facilitating the migration and destruction of deeper layers of the corneal epithelium suggesting that the contact-dependent activity is an important pathogenic mechanism of *Acanthamoeba castellanii* and *Acanthamoeba polyphaga*.

## Figures and Tables

**Table 1:** Summary of pathogenicity tests of *A. castellanii* and *A. polyphaga* in a murine model according to Culbertson et al. [19].

	% Virulence	Amoebae recovery	Dead postinoculation
*A. castellanii *	60%	Brain, lung, kidney, liver	5–9 days
*A. polyphaga *	20%	Brain, lung	7–20 days

**Table 2:** Summary of proteolytic activities detected through SDS-PAGE gels copolymerized with gelatin in total extracts (TE) and conditioned medium (CM) from of *A. castellanii* and *A. polyphaga.* The majority of proteases were inhibited 100% with PMSF (1 mM), only two of them were partially inhibited (*).

	Proteases (kDa)	100% inhibition of proteases PMSF (1 mM)
	TE, CM 120	CM 39*
	CM 97	
	TE, CM 66	
	TE, CM 64	
	CM 57	
*A. castellanii *	CM 55	
	CM 50	
	CM 47	
	CM 45	
	CM 40	
	CM 38	

	TE, CM 120	
	CM 97	
	CM 66	
	CM 64	
*A. polyphaga *	CM 57	
	TE 55	TE 58*
	CM 50	
	CM 40	
	CM 38	
